# Epitope specificity of anti-synapsin autoantibodies: Differential targeting of synapsin I domains

**DOI:** 10.1371/journal.pone.0208636

**Published:** 2018-12-13

**Authors:** Robert Mertens, Sarah Melchert, Daniel Gitler, Morten Brix Schou, Sverre Georg Saether, Arne Vaaler, Johannes Piepgras, Elena Kochova, Fabio Benfenati, Gudrun Ahnert-Hilger, Klemens Ruprecht, Markus Höltje

**Affiliations:** 1 Institute of Integrative Neuroanatomy, Charité –Universitätsmedizin Berlin, corporate member of Freie Universität Berlin, Humboldt-Universität zu Berlin, and Berlin Institute of Health, Berlin, Germany; 2 Department of Physiology and Cell Biology, Faculty of Health Sciences and Zlotowski Center for Neuroscience, Ben-Gurion University of the Negev, Beer-Sheva, Israel; 3 Department of Psychiatry, St. Olav’s University Hospital, Trondheim, Norway; 4 Department of Mental Health, Norwegian University of Science and Technology, Trondheim, Norway; 5 Center for Synaptic Neuroscience and Technology, Istituto Italiano di Tecnologia, Genova, Italy; 6 Department of Neurology, Charité –Universitätsmedizin Berlin, corporate member of Freie Universität Berlin, Humboldt-Universität zu Berlin, and Berlin Institute of Health, Berlin, Germany; Ludwig-Maximilians-Universitat Munchen, GERMANY

## Abstract

**Objective:**

To identify the specific domains of the presynaptic protein synapsin targeted by recently described autoantibodies to synapsin.

**Methods:**

Sera of 20 and CSF of two patients with different psychiatric and neurological disorders previously tested positive for immunoglobulin (Ig)G antibodies to full-length synapsin were screened for IgG against synapsin I domains using HEK293 cells transfected with constructs encoding different domains of rat synapsin Ia. Additionally, IgG subclasses were determined using full-length synapsin Ia. Serum and CSF from one patient were also screened for IgA autoantibodies to synapsin I domains. Sera from nine and CSF from two healthy subjects were analyzed as controls.

**Results:**

IgG in serum from 12 of 20 IgG synapsin full-length positive patients, but from none of the healthy controls, bound to synapsin domains. Of these 12 sera, six bound to the A domain, five to the D domain, and one to the B- (and possibly A-), D-, and E-domains of synapsin I. IgG antibodies to the D-domain were also detected in one of the CSF samples. Determination of IgG subclasses detected IgG_1_ in two sera and one CSF, IgG_2_ in none of the samples, IgG_3_ in two sera, and IgG_4_ in eight sera. One patient known to be positive for IgA antibodies to full-length synapsin had IgA antibodies to the D-domain in serum and CSF.

**Conclusions:**

Anti-synapsin autoantibodies preferentially bind to either the A- or the D-domain of synapsin I.

## Introduction

By using a combination of immunoprecipitation methods, mass spectrometry, immunohistochemistry of brain sections from wild type and synapsin-knock mice, as well as cell-based assays and biochemistry we recently identified the presynaptic vesicle-associated protein synapsin as a novel target of antineuronal autoantibodies in serum and CSF samples from a patient with limbic encephalitis [1]. In a follow-up study using rat and human synapsin I transfected HEK293 cells as well as biochemical analyses, serum IgG to full-length rat synapsin Ia could be detected in 23 of 375 (6.1%) patients with different neurological and psychiatric diseases, including patients with psychotic, bipolar or depressive disorders and multiple sclerosis (MS) or clinically isolated syndrome (CIS), but not in 97 healthy controls [2]. Concomitant serum autoantibodies, mostly low titer anti NMDAR antibodies, were detected in eight of the patients.

**Fig 1:**
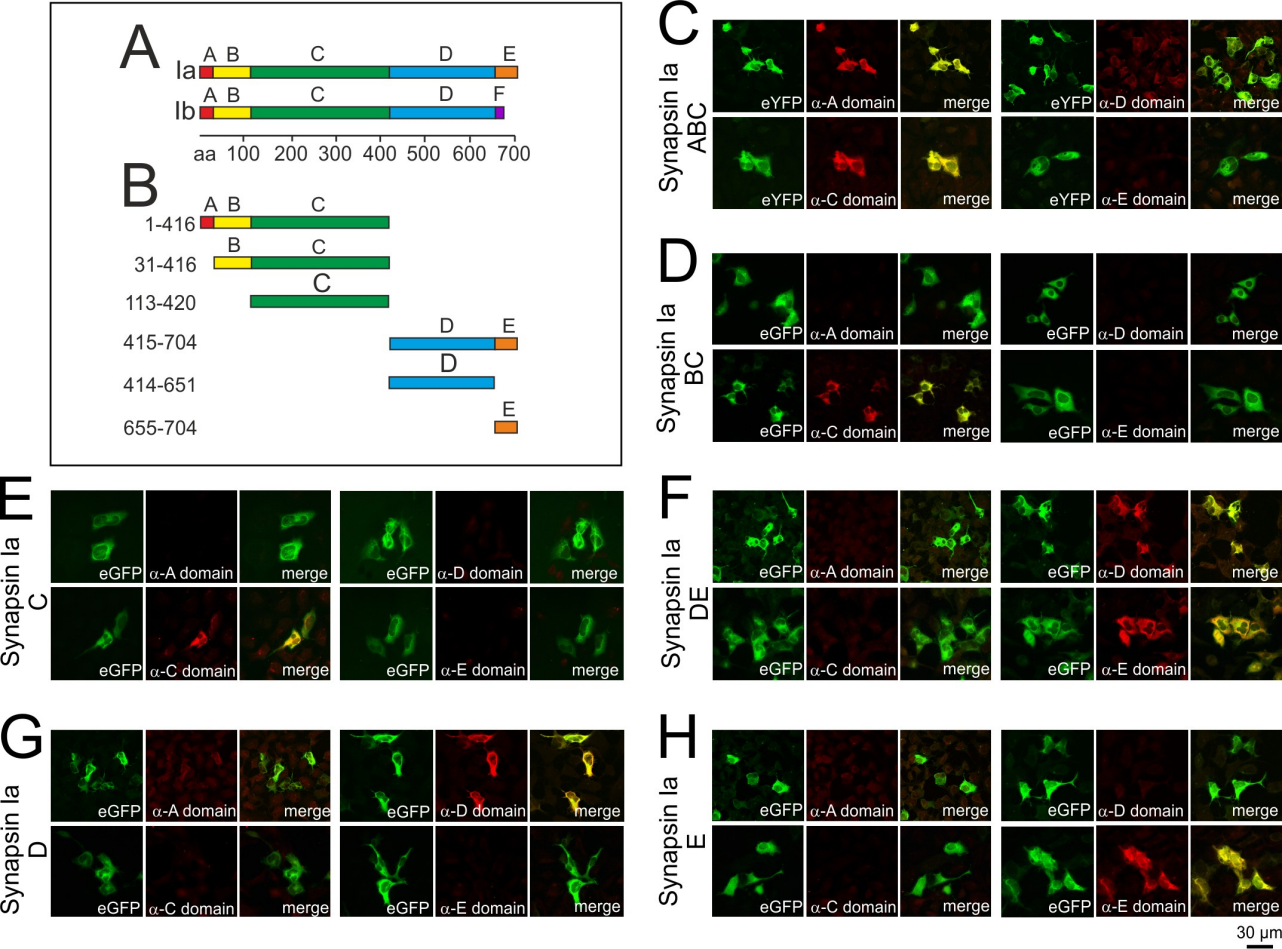
Specific recognition of synapsin I domains expressed in transfected HEK cells by domain-specific antibodies. (A) Domain structure of the synapsin isoforms Ia and Ib. The N-terminal domains A-C are highly conserved in all synapsins. The C-terminal region is variable due to heterogeneous combinations of domains. (B) Diagram of the synapsin Ia fragments examined to investigate targeting of patient synapsin autoantibodies. (C-H) HEK293 cells were transfected with rat synapsin Ia constructs, comprising the eYFP-tagged fragment ABC (C), eGFP-tagged BC (D), eGFP-tagged C (E), eGFP-tagged DE (F), eGFP-tagged D (G), or eGFP-tagged E domain (H). Cells were fixed, permeabilized and incubated with antibodies either directed against the A-domain of synapsin I and II (rabbit anti-synapsin A; dilution 1:500), the D-domain of synapsin I (mouse anti-synapsin D; dilution 1:500), the C-domain of synapsin I/II (rabbit anti-synapsin C; dilution 1:500), or the E-domain of synapsin Ia (rabbit anti-synapsin E; dilution 1:200). Antibody binding was visualized using Alexa Fluor 549-coupled secondary antibodies. Transfected cells were correctly recognized by the respective antibodies that colocalized with the signal of the tags, confirming domain identities and detectability of the various domains by anti-synapsin antibodies.

Synapsin I and II are major synaptic vesicle proteins of the adult brain, regulating the availability of synaptic vesicles and thus affecting synaptic transmission [3; 4; 5]. Synapsins are composed of different highly preserved domains that were defined by sequence homology across species [6]. Synapsin Ia and Ib are two splice variants, which share the A-, B-, C- and D-domains, but differ in the C-terminal region, consisting of the E domain in synapsin Ia and the shorter F-domain in synapsin Ib (see also Fig 1A). Distinct functional roles, such as interference with the generation of excitatory postsynaptic potentials attributed to domain A of synapsin I, were ascribed to individual domains of synapsin I [7]. Membrane insertion involves domain C of synapsin I [8] and interaction with SH3 domain-containing endocytic proteins was shown for the D-domain of synapsin I [9].

The pathophysiological relevance of antibodies to synapsin remains to be clarified. Our finding that antibodies to synapsin are associated with different clinical phenotypes raises the question whether this may be related to distinct functions of synapsin and to the recognition of different domains of synapsin by autoantibodies to synapsin. To better understand the potential pathophysiological relevance of antibodies to synapsin, we here characterized in molecular detail the specific domains of synapsin targeted by synapsin autoantibodies.

## Materials and methods

### Ethical approval

The study was approved by the institutional review board of Charité– Universitätsmedizin Berlin (EA1/182/10; EA1/129/14, EA1/096/12) and the regional committee for medical and health research ethics, central Norway (2011/137). All participants provided written informed consent.

### Patients

Patient sera investigated in this study were previously shown to contain antibodies to full-length rat synapsin Ia and included sera from 16 patients positive for IgG autoantibodies to rat synapsin Ia identified from a total of 207 patients admitted to acute psychiatric inpatient care at the Department of Psychiatry, St. Olav’s University Hospital, Trondheim, Norway, between September 2011 and March 2012 [10]. Additionally, three sera and 1 CSF from three anti-synapsin IgG positive patients with a clinically isolated syndrome (CIS) were available from a cohort total of 78 patients with a CIS or early relapsing-remitting MS (RRMS) collected at the NeuroCure Clinical Research Center/Department of Neurology, Charité –Universitätsmedizin Berlin. The patients’ demographic and clinical findings were recently described in detail [2]. In addition, we analyzed serum and cerebrospinal fluid (CSF) from the 69-year old male index patient diagnosed with limbic encephalitis in 2013 in whom IgA and IgG autoantibodies to synapsin I and II were originally identified [1]. As controls, nine sera and two CSF samples from healthy subjects were obtained at the NeuroCure Clinical Research Center/Department of Neurology, Charité –Universitätsmedizin Berlin.

### Synapsin plasmids

The expression constructs for the various synapsin domains have been described earlier [6]. In brief, domain cDNAs were cloned into either pEGFP or pEYFP C1-C3 vectors. The following domains or domain combinations constituting sequence fragments of rat synapsin Ia (UniProt/SWISS-PROT AAA42145.1) were used in our study: pEYFP-ABC; pEGFP-BC; pEGFP-C; pEGFP-D; pEGFP-DE and pEGFP-E. From the sequence of rat synapsin Ia, the following fragments were constructed: these constructs contained the N-terminal ABC fragment (aa 1–416), the BC fragment (aa 31–416), the C-domain (aa 113–420), the DE fragment (aa 415–704), the D-domain (aa 414–651) and the E-domain (aa 655–704) (for detail see also Fig 1A, B). Rat full-length synapsin Ia cDNA was tagged on the N-terminus with enhanced green fluorescent protein (eGFP) based on a pEGFP-C1 backbone.

### Primary antibodies

A monoclonal mouse antibody against the D-domain of synapsin I (#106011) and a polyclonal rabbit antibody against the A-domain of synapsin I/II (#106002) were purchased from Synaptic Systems (Göttingen, Germany). Rabbit polyclonal antibodies against the C-domain of synapsin I/II and the E-domain of synapsin Ia were developed by one of us (F.B.), as described earlier [11]. A guinea pig polyclonal antibody against GFP used in some experiments to enhance endogenous fluorescence was also obtained from Synaptic Systems (#132005).

### Secondary antibodies

Mouse antibodies were visualized using Alexa Fluor 594 goat anti-mouse IgG (MoBiTec, Göttingen, Germany; # A11032). An Alexa Fluor 488 goat anti-guinea pig IgG (#A11073) was also from MoBiTec. Alexa Fluor 594 goat anti-rabbit IgG (#A11037; Life Technologies, Carlsbad, CA, USA) was applied to detect bound rabbit antibodies. Bound human antibodies of the immunoglobulin type G (total) were visualized by incubation with Alexa Fluor 594 labeled goat anti-human IgG directed against the Fc fragment (Dianova GmbH, Hamburg, Germany; #109-585-008). Visualization of human IgG subclass-specific antibodies was achieved using Alexa Fluor 647 labeled goat anti-human IgG_1_, IgG_2_, IgG_3_ and IgG_4_ antibodies (Southern Biotech, Birmingham, USA; #9052–31, #9070–31, #9210–31, #9200–31). Detection of immunoglobulin A antibodies in the patients’ CSF or serum was performed by using a Texas Red 594 coupled goat anti-human IgA secondary antibody (Dianova; #109-076-011).

### Cell-based assays for synapsin domains and full-length synapsin Ia

Human embryonic kidney (HEK) 293 cells were cultured in 24-well multiplates to 70% confluency and transfected with 0.5–1 μg plasmid cDNA per well for 24 hours using Lipofectamine (Gibco/BRL Life Technologies, Eggenstein, Germany). Transfected cells were fixed with ice-cold 80% methanol for 20 minutes at -20°C (or 4% paraformaldehyde at 4°C for detection of IgA immunoreactivity). Cells were subsequently permeabilized with 0.1% Triton X-100. Thereafter, cells were incubated with either the domain specific primary antibodies at the indicated concentrations or with patient or control serum or CSF at a dilution of 1:320.

### Antibody index calculation

HEK 293 cells transfected with rat synapsin Ia were processed as described above and incubated with increasing dilutions (1:320, 1:1000, 1:3200, 1:10.000, and 1:32,000) of patient CSF or serum to determine titers of anti-synapsin IgG in a paired CSF/serum sample. Alexa Fluor 594 goat anti-human IgG served as secondary antibody. Antibody index (AI) for IgG antibodies to synapsin Ia was calculated as previously described by the formula: AI = (anti-synapsin titer CSF^-1^/anti-synapsin titer serum^-1^)/(total Ig CSF/total Ig serum). AI values above four were considered as evidence of an intrathecal antibody synthesis [12].

## Results

### Cell-based assays for detection of antibodies to synapsin I domains

From the sequence of rat synapsin Ia, six different combinatory domain fragments or single domains were constructed (Fig 1A and 1B). A rationale to use fragments from rat synapsin sequence was the fact that these constructs have been extensively characterized for targeting and binding to presynaptic terminals [6]. Of note, human and rat synapsin I share a 95% identity of the primary structure [13] and binding of patient antibodies to rat and human protein exhibited a very similar pattern [2].

To establish cell-based assays for the detection of antibodies to synapsin I domains, we transfected six different rat synapsin Ia constructs (Fig 1B) in human embryonic kidney (HEK) 293 cells. Subsequently, transfected cells were incubated with antibodies specifically recognizing the A-, C-, D- or E-domain of synapsin I.

As shown in Fig 1C–1H all six synapsin I fragments were clearly detectable by their endogenous fluorescence tags (eYFP for the ABC-fragment and eGFP for all other constructs). Furthermore, all individual or combined synapsin Ia domains were specifically recognized by antibodies directed against the respective domains indicating proper assay function.

### Antibodies to synapsin predominantly target the A- or D-domain of synapsin Ia

To decipher the domain specificity of autoantibodies to synapsin Ia, we analyzed a total of 20 serum and two CSF samples, which contained antibodies to full-length rat synapsin Ia as identified in our previous studies [1; 2]. For control, nine sera and two CSF samples from healthy controls were analyzed as well.

We first screened sera and one CSF from 19 patients positive for rat synapsin IgG for their reactivity with the various synapsin domains. Sixteen of these patients were diagnosed with psychiatric disorders and three patients with a CIS. CSF was available from one of the patients with a CIS. A summary of the findings is presented in Table 1.

**Table 1:** Demographic, antibody specificity and clinical findings of the 13 patients with serum or CSF antibodies to synapsin Ia fragments identified in this study.

Serum code *Höltje et al*., *2017*	Sex	Age (years)	ABC	BC	C	DE	D	E	Synapsin Ia full length titer	Diagnoses
Index patient serum	m	69	-	-	-	+ (IgA)	+ (IgA)	-	1:1000–1:3200	Limbic encephalitis
Index patient CSF	„	„	-	-	-	+ (IgA)	+ (IgA)	-	1:1000–1:3200	„
CIS_0036	f	34	-	-	-	-	-	-	1:3200	Clinically isolated syndrome; panic/anxiety and recurrent depressive disorder
CIS_0064	f	51	-	-	-	-	-	-	1:1000	Clinically isolated syndrome
CIS_0117 serum,	f	24	-	-	-	+	+	-	1:10000	Clinically isolated syndrome
CIS_0117 CSF	„	„	-	-	-	+	+	-	1:1000	„
PSY_0012	f	29	+	-	-	-	-	-	1:320	Bipolar affective disorder type 1, mixed episode
PSY_0032	f	20	+	-	-	-	-	-	1:320	Bipolar affective disorder type 2, depressive episode
PSY_0045	f	32	-	-	-	-	-	-	1:1000	Persistent personality change after catastrophic experience
PSY_0068	f	52	-	-	-	+	-	-	1:10000	Recurrent depressive disorder, severe episode; coma for 12 days at age 8 years after accident
PSY_0082	m	26	-	-	-	-	-	-	1:320	Acute polymorphic psychotic disorder; Möbius syndrome
PSY_0094	m	39	-	-	-	-	+	-	1:100000	Alcohol abuse, ADHD, bipolar affective disorder type 2, emotionally unstable personality disorder
PSY_0141	f	21	-	-	-	+	+	-	1:1000	Alcohol dependency syndrome, ADHD, PTSD, mixed obsessional thoughts and acts
PSY_0149	m	51	+	+	-	+	+	+	1:100000	Bipolar affective disorder type 1, manic episode; alcohol dependency syndrome; hepatitis C
PSY_0171	f	22	-	-	-	+	+	-	1:32000	Moderate depressive episode; SLE
PSY_0183	f	31	-	-	-	-	-	-	1:3200	Recurrent depressive disorder, moderate episode; personality disorder
PSY_1851	f	58	+	-	-	-	-	-	1:1000	Bipolar disorder type 1, manic episode with psychotic symptoms; MS; later diagnosed organic affective disorder
PSY_1852	m	68	+	-	-	-	-	-	1:320	Bipolar affective disorder type 1, manic episode; paranoid psychosis
PSY_0189	f	77	+	-	-	-	-	-	1:3200	Recurrent depressive disorder
PSY_0201	f	44	-	-	-	-	-	-	1:1000	Recurrent depressive disorder, moderate episode; alcohol dependency syndrome; epileptic seizures as child; probably anoxic injury during birth
PSY_0203	m	27	+	-	-	-	-	-	1:320	Paranoid schizophrenia; multiple drug abuse
PSY_0204	m	60	-	-	-	-	-	-	1:1000	Recurrent depressive disorder, mild episode; cannabinoid abuse; melanoma; hepatitis C; psoriasis

Presence (+) or absence (-) of immunoreactivity is indicated.

Diagnoses, antibody titers to full length synapsin and concomitant autoantibodies (not shown) have been published previously [2].

ADHD = attention deficit hyperactivity disorder; f = female; m = male; MS = multiple sclerosis; PTSD = post traumatic stress disorder; SLE = systemic lupus erytematodus

**Fig 2:**
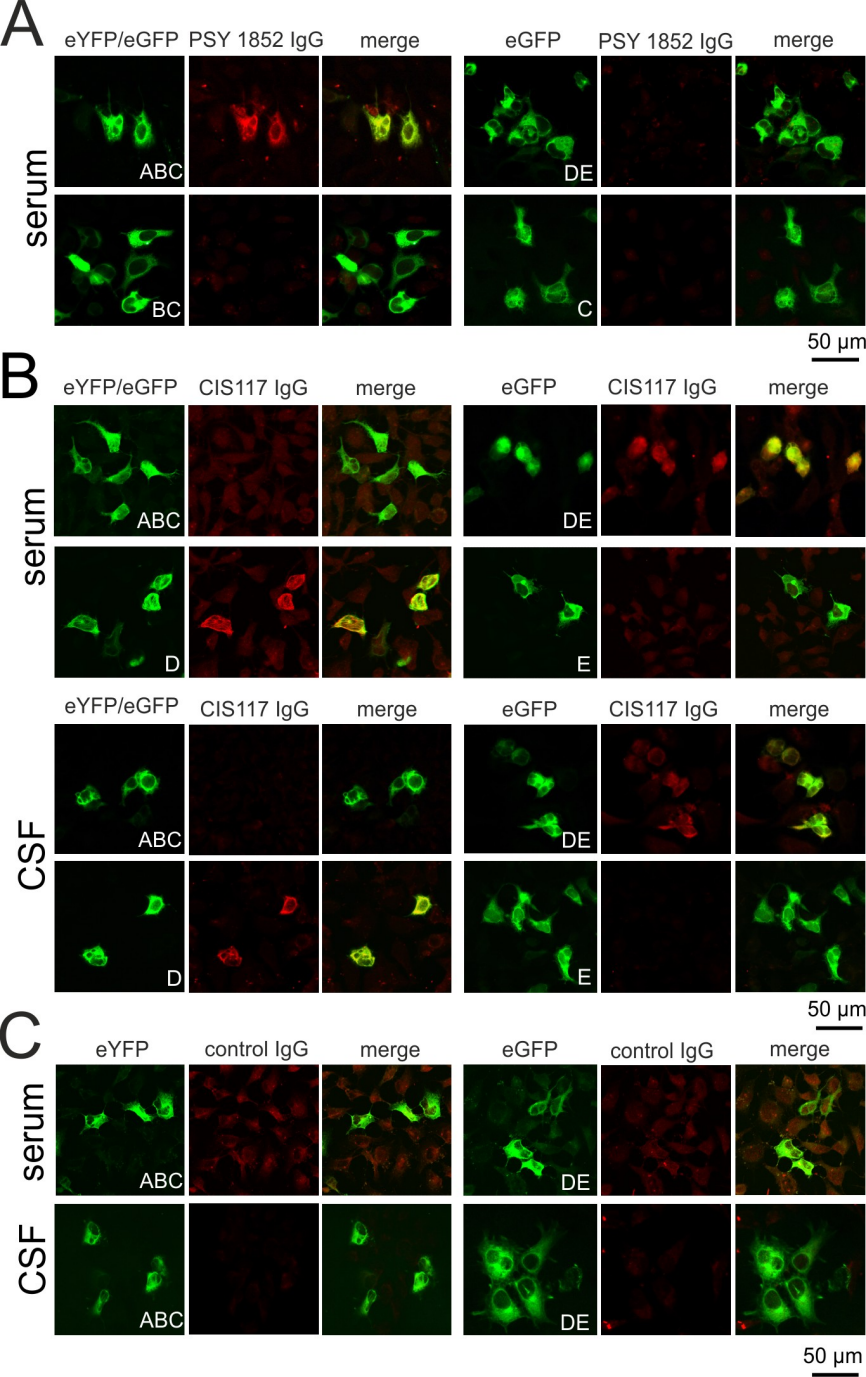
Serum IgG autoantibodies against the A-domain of a patient with bipolar affective disorder and serum and CSF IgG autoantibodies against the D-domain of a patient with a clinically isolated syndrome (CIS). (A) HEK293 cells were transfected with eYFP-tagged synapsin I fragment ABC, eGFP-tagged DE, BC or C. Cells were incubated with patient serum at a dilution of 1:320. Bound IgG was detected using an Alexa 594-coupled secondary antibody to the Fc fragment of human IgG. The patient’s serum demonstrated positive immunoreactivity that co-localized with the eYFP tag of synapsin ABC, but with none of the other constructs. (B) HEK293 cells were transfected with eYFP-tagged synapsin I fragment ABC, eGFP-tagged DE, D or E. Cells were incubated with patient serum or CSF at a dilution of 1:320. Bound IgG was detected using an Alexa 594-coupled secondary antibody to the Fc fragment of human IgG. Both the patient’s serum and CSF demonstrated positive immunoreactivity that co-localized with the eGFP tag of synapsin DE and the single D-domain, but with none of the other constructs. (C) Serum and CSF of two healthy controls (control IgG) did not bind to cells transfected with synapsin ABC or DE.

Out of the sera of 16 patients with psychiatric disorders positive for antibodies to full-length synapsin, 11 sera exhibited a detectable immunoreactivity against one or more of the synapsin Ia domains. Out of the three patients diagnosed with a CIS, serum and CSF from one patient showed immunoreactivity to synapsin Ia domains. Among these 12 positive sera, seven were positive for the ABC fragment (58,3%). Among these seven sera, only one serum was also positive for the BC fragment and none was positive for the single C-domain. Thus, sera of six patients were likely exclusively positive for the A-domain (as exemplarily shown for one patient in Fig 2A) and one proved to be positive for both the A- and B-domains, or solely the B-domain (PSY_0149, the only patient with multiple targeted domains, Table 1). A direct approach towards testing reactivity to the A- or B-domain alone was not possible due to the lack of individual constructs for these domains. The only serum that reacted with both the ABC and the BC fragment also reacted with the C-terminal domains D and E.

The five sera (41,7%) that did not react with the ABC fragment exhibited reactivity to either the DE fragment and the D-domain (three), the DE fragment alone (one) or the D-domain alone (one). None of these five sera showed reactivity towards the E-domain alone, indicating that the D-domain was the targeted antigenic region of synapsin Ia. A CSF sample available from a patient whose serum reacted with the DE fragment and the D-domain likewise reacted with the DE fragment and the D-domain. The results of the cell-based assays for this patient with a CIS are shown in Fig 2B. For this patient, antibody titers of the serum/CSF pair were determined using full-length synapsin Ia and the antibody index (AI) was calculated. The AI value of 19,6 clearly indicated an intrathecal synthesis of IgG to synapsin I. Additionally, all nine control sera and two CSF samples applied at the same dilution as the patients’ sera were negative for IgG synapsin autoantibodies, as exemplarily shown for two healthy subjects in Fig 2C.

Of note, all patient and control samples were also tested for IgA reactivity to the ABC and DE fragments. All samples remained negative, thereby confirming our previous results obtained for full-length synapsin [2]. As a further control, 10 patient sera from that study negative for antibodies to full-length synapsin were tested for IgG and IgA reactivity to the ABC or DE fragment. Also in this case no reactivity to one of the fragments was detected.

### IgA antibodies from the index patient with limbic encephalitis target the D-domain of synapsin Ia

**Fig 3:**
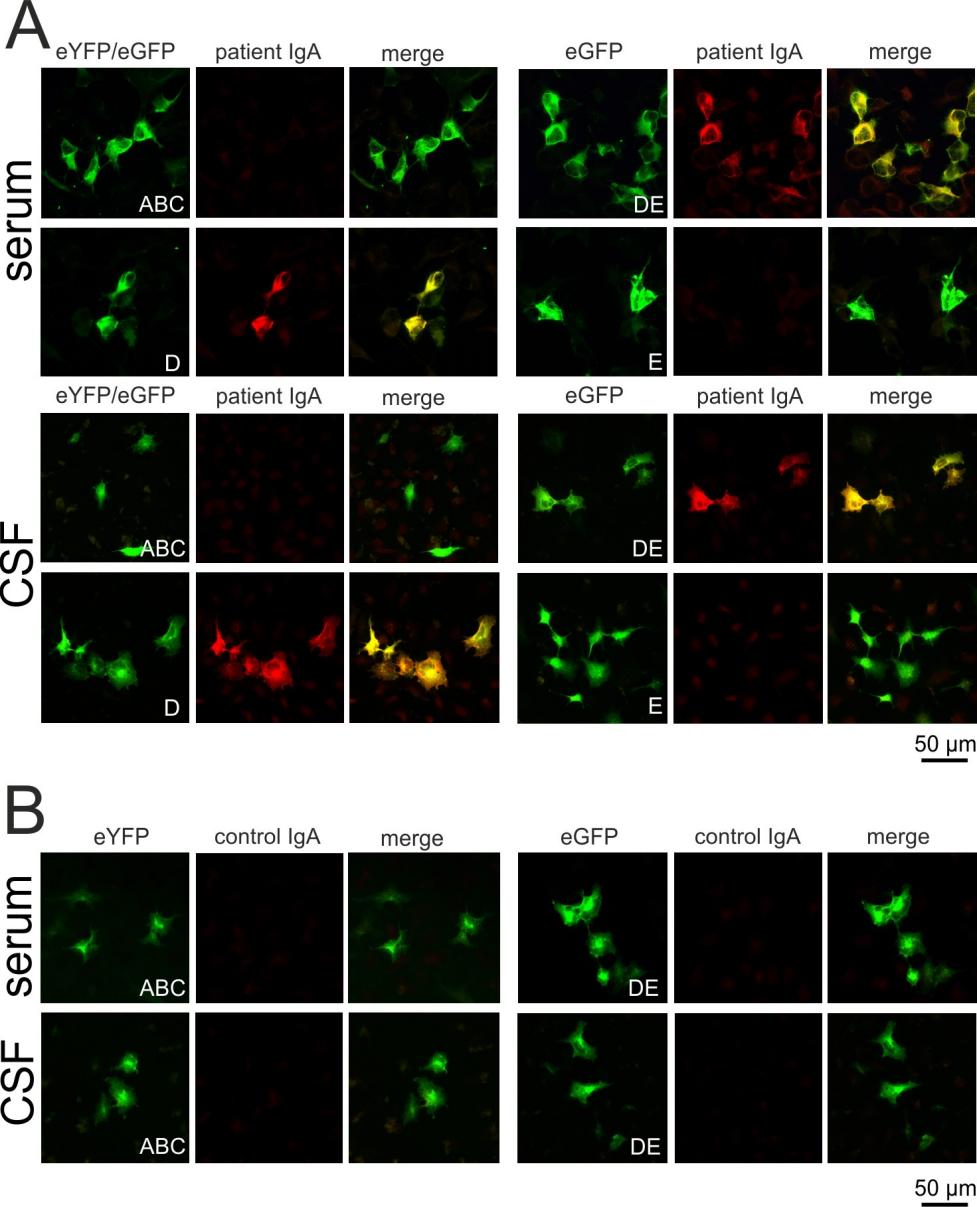
IgA antibodies to synapsin in serum and CSF of the previously described index patient with limbic encephalitis are directed against the D-domain of synapsin. (A)HEK293 cells were transfected with eYFP-tagged synapsin I fragment ABC, eGFP-tagged DE, D or E. Cells were incubated with patient serum or CSF at a dilution of 1:320. Bound patient IgA was detected using a Texas red-coupled secondary antibody directed against human IgA. Both the patient’s serum and CSF (Patient IgA) demonstrated positive immunoreactivity that co-localized with the eGFP tag of synapsin DE and single D fragments, but with none of the other constructs. (B) Serum and CSF of two healthy controls (control IgA) did not bind to the cells transfected in the same way.

We also analyzed serum and CSF from our index patient, who had high titers of anti-synapsin IgA and IgG in serum and CSF [1]. Both serum and CSF IgA clearly reacted with the DE fragment and the D-domain, but not with the ABC or E construct (Fig 3A). Of note, we could not detect reactivity of IgG antibodies to any of the domains despite the fact that serum and CSF IgG reacted with full-length synapsin. Serum and CSF IgA from two healthy subjects did not react with any of the synapsin constructs (Fig 3B).

**Fig 4:**
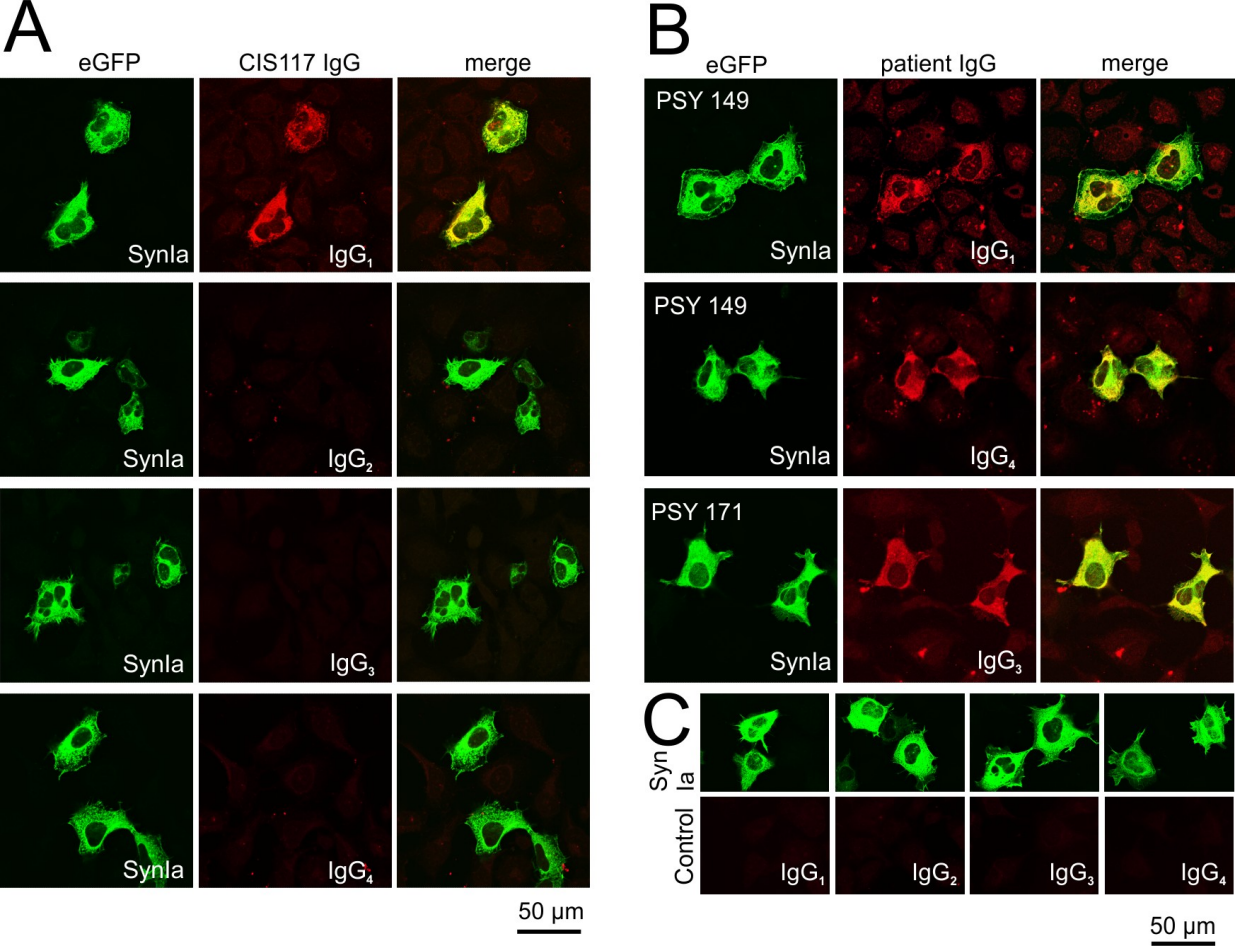
IgG subclasses of antibodies to synapsin in sera of patients with a clinically isolated syndrome (CIS), bipolar disorder and depression. (A) HEK293 cells were transfected with eGFP-tagged full length rat synapsin Ia and incubated with serum of patient CIS117 at a dilution of 1:320. Bound antibodies of the IgG subclasses IgG_1-4_ were detected using subclass-specific Alexa 647-coupled secondary antibodies. The patient’s serum demonstrated positive immunoreactivity for IgG_1_ antibodies but none of the other subclasses. (B) HEK293 cells were transfected with eGFP-tagged full length rat synapsin Ia and incubated with serum of patient PSY 149 (upper two panels) and patient PSY 171 at a dilution of 1:320. Bound antibodies of the IgG subclasses IgG_1-4_ were detected as given in (A). Serum of patient PSY 149 demonstrated positive immunoreactivity for IgG_1_ and IgG_4_ antibodies but none of the other subclasses. Serum of patient PSY 171 demonstrated positive immunoreactivity for IgG_3_ antibodies but none of the other subclasses. (C) Serum of a healthy control did not exhibit immunoreactivity for any of the IgG subclasses.

After successful determination of domain specificities we additionally aimed to identify the respective IgG subclasses involved in autoimmunity against synapsin. To this end, we transfected HEK 293 cells with full-length synapsin Ia, again incubated fixed cells with patient sera that reacted with any of the fragments as well as with the CSF of CIS117 at a dilution of 1:320 and applied secondary antibodies specific for the four subclasses IgG_1,_ IgG_2_, IgG_3_ and IgG_4_ (Fig 4A–4C). As a result, 1 serum and the corresponding CSF exclusively contained IgG_1_, 2 sera exclusively showed IgG_3_ reactivity, 7 sera only showed IgG_4_ reactivity, and one serum was positive for IgG_1_ and IgG_4._ No IgG_2_ reactivity was observed (for summary, see Table 2). Noteworthy, the patient with two IgG subclasses was identical with the patient that exhibited immunoreactivity to more than one domain. One single serum (patient PSY_203) yielded no clearly interpretable results and was therefore excluded from the summary in Table 2. Overall, no strict correlation of one of the subclasses with a single domain was observed.

**Table 2:** Distinct IgG subclasses of antibodies to synapsin Ia.

Patient	IgG_1_	IgG_2_	IgG_3_	IgG_4_	Target Domain
CIS117 (serum)	+	-	-	-	D
CIS117 (CSF)	+	-	-	-	D
PSY_0012	-	-	-	+	A
PSY_0032	-	-	-	+	A
PSY_0068	-	-	-	+	D
PSY_0094	-	-	-	+	D
PSY_0141	-	-	-	+	D
PSY_0149	+	-	-	+	A,B,D,E*
PSY_0171	-	-	+	-	D
PSY_1851	-	-	-	+	A
PSY_1852	-	-	-	+	A
PSY_0189	-	-	+	-	A

Presence (+) or absence (-) of immunoreactivity is indicated.

* Differential attribution of IgG_1_ and IgG_4_ antibodies to individual domains is unknown.

### Demographical data of patients with synapsin autoantibodies

The diagnoses, clinical and paraclinical findings of all synapsin-positive patients have been published in our previous studies. In the current study 13 (eight females, five males) out of 20 patients exhibited immunoreactivity to fragments of synapsin Ia and had a mean ± SD (range) age of 42,9 ± 19,8 (20–77) years. Controls (nine) had a mean ± SD (range) age of 38,6 ± 17,2 (18–65) years.

## Discussion

Despite the apparent clinical heterogeneity, a preference in epitope specificity of anti synapsin autoantibodies in psychiatric and neurological patients exists. Of the 13 patients showing a clearly detectable immune reaction to one of the investigated synapsin fragments six sera exhibited binding of IgG to the A-domain. The same number was detected for the D-domain. Binding to the B-domain and E-domain was shown for one individual patient. Concerning IgG subclasses, IgG_1_, IgG_3_ and, with highest prevalence, IgG_4_ were detected.

### Intracellular synaptic proteins as autoimmune targets: Pathogenic relevance

Given the fact that synapsin is a ubiquitous presynaptic protein, occurrence of autoantibodies in a variety of psychiatric and neurological disorders is conceivable. Subject to functional evidence, it remains unclear if the existence of autoantibodies to synapsin in serum or CSF has a pathogenic relevance or represents an epiphenomenon. In addition, it remains to be elucidated whether the antigenic epitope presentation of synapsin domains relies on the extracellular presentation of synapsin in the course of an ongoing neuronal repair mechanism as proposed in an *in vivo* model [14], a specific extracellular presentation by a so far unknown surface transport process or just results from neuronal cell loss. Irrespective of how the immune system gets access to synapsin, an intracellular action of antibodies might occur, as shown for autoantibodies against neurofilament proteins [15]. In contrast to other known autoantibodies to intracellular target proteins such as anti-Hu, anti-Ri or anti-Yo, [16; 17; 18] no strong tumor association was detectable in patients with autoantibodies to synapsin. Moreover, the index patient clinically improved following immunotherapy indicating a functional relevance of anti synapsin antibodies [1]. Hitherto, autoantibodies to intracellular synaptic proteins other than synapsin have already been described for glutamate decarboxylase 65 (GAD65) and amphiphysin in large patient cohorts [19; 20]. For these two autoantibodies associated with different neurological diseases like stiff-man syndrome evidence for a pathogenic potential was suggested by rodent animal models showing induction of anxiety by transfer of stiff-person IgG [21] or a reduced GABAergic inhibition by amphiphysin IgG [22]. Internalisation of amphiphysin antibodies was shown recently to reduce the presynaptic vesicle pool, adding to mechanistic understanding of a suggested pathogenic effect [23]. In the case of synapsin, a putative pathogenic mechanism of antibody binding might rely on the interference with the phosphorylation status of synapsin, a modification crucially involved in regulating its function and shown to affect residues both of the A and the D domain regions [24].

When comparing the clinical diagnoses, antibodies to the D-domain were observed in a somewhat broader spectrum of diseases (depression, bipolar disorder, ADHD, CIS, limbic encephalitis) than antibodies to the A-domain, that dominated in bipolar disorder patients (four out of six patients). The relatively small number of patients, however, prevents us from drawing definite conclusions on the association of specific diseases with antibodies to distinct synapsin domains.

In addition to our previous prevalence study that addressed the presence of antibodies in serum we had one additional CSF sample available for testing (CIS_0117). As shown for IgA antibodies of the index patient with limbic encephalitis this CSF also showed IgG reactivity to the D-domain. Future studies are needed to show whether the D-domain is the preferred target of CSF antibodies. Noteworthy, for both available CSF samples with antibodies to synapsin an intrathecal synthesis of immunoglobulins could be detected in this study and [1].

### Antibodies to full length synapsin not reacting with single domains or domain stretches

The observation that seven out of 20 sera reacting with full-length synapsin Ia did not react with distinct synapsin domains might be explained by the fact that a certain conformational epitope structure can be provided only by the full-length protein. Missing homology between human and rat sequences can be ruled out to account for this observation since all 20 sera reacted with full length rat synapsin. Alternatively, anti synapsin antibodies may exhibit a lower affinity to the expressed domains than to the full-length protein and therefore escaped detection in our assay, that was standardized to yield a high signal to noise ratio based on a 1:320 dilution of the applied patient samples. Antibody titers to full length protein were initially analysed for all individual sera [2] but can not be used to predict the binding probability to individual domains since sera with a titer range of 1:320–1:100.000 bound to synapsin fragments. Folding differences between the individual synapsin constructs might also account for the observation that two sera exclusively reacted with either the DE fragment or the D-domain but not with both constructs as expected.

### Distinct IgG subclasses

Of the four IgG subclasses described in the literature we were able to detect IgG_1_ (two patients), IgG_3_ (two patients) and, most frequently, IgG_4_ (eight patients) in our cohort. Despite being the last prevalent subclass detected in serum [25] IgG_4_ antibodies therefore seem to be the dominant, albeit not exclusive, subclass in synapsin autoimmunity, at least for the relatively few cases described so far. So, the ability to fix complements seems not to be necessary for the immune reaction in the majority of patients. Antibodies of the IgG_4_ subclass have already been described for LGI1 and Caspr2 encephalitis [26; 27], and DPPX encephalitis [28]. Moreover, this subclass is associated with an IgG_4_-related systemic disease, able to cause pancreatitis and infection of other organs [29, 30].

In summary, the present study adds data to further characterize the recently identified antineuronal antibodies against synapsin in molecular detail. Functional cell culture and animal studies investigating the pathological potential of these antibodies by electrophysiological and biochemical methods are under way to shed light on the putative pathogenic impact of these antibodies.
